# DOES PERTURBATION-BASED BALANCE TRAINING ON COMMERCIAL TREADMILLS IMPROVE BALANCE RECOVERY IN OLDER ADULTS?

**DOI:** 10.1093/geroni/igac059.144

**Published:** 2022-12-20

**Authors:** Justin Whitten, Bryant Oleary, Dawn Tarabochia, David Graham

**Affiliations:** Montana State University, BOZEMAN, Montana, United States; Montana State University, Bozeman, Montana, United States; Montana State University, Bozeman, Montana, United States; Montana State University, Bozeman, Montana, United States

## Abstract

**Background:**

Perturbation-based training (PBT) is a balance training method that causes a trip like event requiring a rapid step response to regain balance. There are numerous examples in the literature demonstrating the effectiveness of PBT but the need to use an expensive treadmill in a scientific laboratory limits the general applicability of PBT as a community-based intervention. A possible solution is to rapidly stop the treadmill belt during walking using the e-brake as the perturbation event. Importantly this could be performed on a commercially available, lower cost treadmill. Therefore, the purpose of this study was to evaluate the effectiveness of a commercial treadmill during PBT.

**Methods:**

Seventeen participants completed either 9 weeks of PBT or conventional balance training based on ACSM guidelines. During an initial and final testing session participants balance recovery performance was evaluated. Participants were released from a forward static lean angle and asked to recover with a single step, during this test their movement was recorded and subsequently used to determine the Margin or Stability pre- and post-training. Participants were tracked for 6 months following the intervention and falls were recorded on a weekly basis. Results and Summary: There was no difference in balance recovery performance between groups following the training intervention and there was no difference in fall rate between groups in the 6-month follow-up period. We conclude that overall using the e-brake of a commercial treadmill is ineffective as a PBT strategy as it elicits no greater benefit than conventional exercise training.

